# Pharmacogenomics Factors Influencing the Effect of Risperidone on Prolactin Levels in Thai Pediatric Patients With Autism Spectrum Disorder

**DOI:** 10.3389/fphar.2021.743494

**Published:** 2021-10-06

**Authors:** Yaowaluck Hongkaew, Andrea Gaedigk, Bob Wilffert, Roger Gaedigk, Wiranpat Kittitharaphan, Nattawat Ngamsamut, Penkhae Limsila, Apichaya Puangpetch, Rattanaporn Sukprasong, Chonlaphat Sukasem

**Affiliations:** ^1^ Division of Pharmacogenomics and Personalized Medicine, Department of Pathology, Faculty of Medicine Ramathibodi Hospital, Mahidol University, Bangkok, Thailand; ^2^ Laboratory for Pharmacogenomics, Somdech Phra Debaratana Medical Center (SDMC), Ramathibodi Hospital, Bangkok, Thailand; ^3^ Research and Development Laboratory, Bumrungrad International Hospital, Bangkok, Thailand; ^4^ Division of Clinical Pharmacology, Toxicology and Therapeutic Innovation, Children’s Mercy Kansas City, Kanas City, MO, United States; ^5^ School of Medicine, University of Missouri-Kansas City, Kansas City, MO, United States; ^6^ Unit of PharmacoTherapy, Epidemiology and Economics, Groningen Research Institute of Pharmacy, University of Groningen, Groningen, Netherlands; ^7^ Department of Clinical Pharmacy and Pharmacology, University of Groningen, University Medical Center Groningen, Groningen, Netherlands; ^8^ Department of Mental Health Services, Yuwaprasart Waithayopathum Child and Adolescent Psychiatric Hospital, Ministry of Public Health, Samut Prakan, Thailand; ^9^ Pharmacogenomics and Precision Medicine, Preventive Genomics and Family Check-up Services Center, Bumrungrad International Hospital, Bangkok, Thailand

**Keywords:** risperidone, prolactin, autism spectrum disorder, genetic risk score, dopamine D2 receptor (DRD2)

## Abstract

We investigated the association between genetic variations in pharmacodynamic genes and risperidone-induced increased prolactin levels in children and adolescents with autism spectrum disorder (ASD). In a retrospective study, variants of pharmacodynamic genes were analyzed in 124 ASD patients treated with a risperidone regimen for at least 3 months. To simplify genotype interpretation, we created an algorithm to calculate the dopamine D2 receptor (*DRD2*) gene genetic risk score. There was no relationship between prolactin levels and single SNPs. However, the H1/H3 diplotype (A2/A2-Cin/Cin-A/G) of *DRD2*/ankyrin repeat and kinase domain containing 1 (*ANKK1*) *Taq*1A, *DRD2* -141C indel, and *DRD2* -141A>G, which had a genetic risk score of 5.5, was associated with the highest median prolactin levels (23 ng/ml). As the dose-corrected plasma levels of risperidone, 9-OH-risperidone, and the active moiety increased, prolactin levels in patients carrying the H1/H3 diplotype were significantly higher than those of the other diplotypes. *DRD2* diplotypes showed significantly high prolactin levels as plasma risperidone levels increased. Lower levels of prolactin were detected in patients who responded to risperidone. This is the first system for describing *DRD2* haplotypes using genetic risk scores based on their protein expression. Clinicians should consider using pharmacogenetic-based decision-making in clinical practice to prevent prolactin increase.

## Introduction

Risperidone is an atypical antipsychotic used to treat autism spectrum disorder (ASD). Its effect is mediated via dopamine D2 receptor and 5-hydroxytryptamine type 2A receptor antagonism (Corena-McLeod, 2015). The US Food and Drug Administration has approved risperidone for the treatment of irritability in children and adolescents aged 5–16 years with ASD (Goel et al., 2018). Risperidone has a more favorable safety and efficacy profile than typical antipsychotic drugs (Peuskens et al., 2014). Two clinical trials (McCracken et al., 2002; Shea et al., 2004) established the efficacy and tolerability of risperidone in patients with ASD and showed that risperidone significantly attenuated disruptive behaviors compared with the placebo over 8 weeks, as measured by a reduction in the irritability subscale of the aberrant behavior checklist (ABC) scores. The improvement in the risperidone treatment group was higher (56.9–64.0%) compared with that in the placebo group (14.1–31.0%) (McCracken et al., 2002; Shea et al., 2004).

Several studies (Baumann et al., 2004; Riedel et al., 2005) have explored the utility of plasma drug monitoring as a biomarker of treatment response. Therapeutic drug monitoring can improve the efficacy and safety of risperidone. Plasma levels of 20–60 ng/L of the active moiety lead to better clinical outcomes in adults with schizophrenia (Baumann et al., 2004; Nazirizadeh et al., 2010). Plasma levels of the active moiety in non-responders were significantly higher than in responders in a 6 weeks clinical trial of risperidone treatment in schizophrenia patients (Riedel et al., 2005). However, no therapeutic drug monitoring data are available for pediatric patients with ASD treated with risperidone.

There is considerable variation in the response to risperidone that may be explained, at least partly, by genetic variation in drug targets. A polymorphism in the dopamine D2 receptor (*DRD2*)/ankyrin repeat and kinase domain containing 1 (*ANKK1*) gene (often referred to as the *Taq*1A polymorphism; rs1800497) was associated with a clinical response in risperidone-treated ASD patients (Nuntamool et al., 2017). The *DRD2*/*ANKK1 Taq*1A A2 or C allele (Sukasem et al., 2016) have been associated with high dopamine receptor densities (Jonsson et al., 1999), which may contribute to blocking dopaminergic activity. *DRD2* -241A>G (rs1799978), located in the 5′-promoter region of *DRD2*, may contribute to increased expression levels. (Nyman et al., 2009). *DRD2* -141C insertion/insertion has been associated with high prolactin levels in antipsychotic-treated male schizophrenia (Zhang et al., 2011). Carriers of the dopamine D3 receptor Gly/Gly allele (rs6280) showed significantly better response rates compared with the Ser/Ser genotype in children with ASD (Firouzabadi et al., 2017). Genetic variation in the 5-hydroxytryptamine type 2A receptor (*HTR2A*), 5-hydroxytryptamine type 2C receptor (*HTR2C*), and ATP binding cassette subfamily B member 1 genes also contribute to clinical outcomes and are possible markers for predicting a positive response to risperidone therapy in ASD (Correia et al., 2010). Moreover, the dopamine transporter [*DAT*; also known as solute carrier family 6 member 3 (SLC6A3)] and serotonin transporter [5-hydroxytryptamine transporter-linked promoter region (*5-HTTLPR*) also known as solute carrier family 6 member 4] genes, although not directly targeted by antipsychotic medications, may influence neurotransmitter availability, and thus contribute to the variability in treatment response (Kirchheiner et al., 2007; Porcelli et al., 2012; Outhred et al., 2016).

The anterior pituitary hormone prolactin has essential physiological functions in the brain. Prolactin acts as a neuropeptide, regulating neuroendocrine and emotional stress responses (Torner, 2016). Serum prolactin levels may also reflect the antipsychotic treatment response. Several clinical studies (Zhang et al., 2002; Ates et al., 2015; Stern et al., 2018) revealed that prolactin may mediate effects on the neuropsychiatric response to risperidone. Zhang et al. (2002) observed a significant positive relationship between the reduction rate of positive subscale scores of the Positive and Negative Syndrome Scale (PANSS) and the change in prolactin levels before and after treatment in chronic schizophrenia. Ates et al. (2015) found that in patients with hyperprolactinemia, the PANSS negative symptom scores were significantly higher than in patients without hyperprolactinemia (*p* = 0.041). Furthermore, several moderators and mediators affecting risperidone response have been described (Stern et al., 2018), and lower baseline levels of prolactin predict responder status in autistic children. Additionally, genetic polymorphisms in the prolactin (*PRL*) (Lee et al., 2007; Ivanova et al., 2017) and prolactin receptor (*PRLR*) (Lee et al., 2007) genes may also contribute to increased prolactin levels. The strongest association was between a single SNP of *PRL* (A>T, rs2244502) and prolactin levels, which showed higher prolactin levels in T carriers than in A carriers. Patients with hyperprolactinemia carried the G/G genotype of −1149 G>T (rs1341239) in the *PRL* gene more frequently than patients without hyperprolactinemia (*p* = 0.009) (Ivanova et al., 2017). Therefore, prolactin is a promising candidate biomarker for risperidone response.

The impact of pharmacogenetics on increased prolactin levels has not been well investigated. Stern et al. (2018) reported the effect of hyperprolactinemia in pediatric ASD patients with disruptive behaviors undergoing risperidone therapy. Therefore, in this study we investigated the association between genetic variations in pharmacodynamic genes and risperidone-induced increases in prolactin levels in children and adolescents with ASD.

## Materials and Methods

### Participants

ASD children and adolescents aged 3–18 years were recruited at the Yuwaprasart Waithayopathum Child Psychiatric Hospital, Samut Prakan, Thailand, in 2017 and 2018. All participants were ethnic Thai. The clinical neuropsychiatric diagnosis was made according to the Diagnostic and Statistical Manual of Mental Disorders, Fifth Edition criteria. The Ethics Committee of the Faculty of Medicine Ramathibodi Hospital, Bangkok, Thailand (MURA2017/556) and Yuwaprasart Waithayopathum Child Psychiatric Hospital, Samut Prakan, Thailand approved the study. All participants or parents of the children signed an informed assent or consent after the study objectives and procedures were explained. Sociodemographic data (gender, age at assessment, daily risperidone dosage, duration of risperidone treatment, and concomitant medication) were collected with a questionnaire. Patients were excluded if they were receiving concomitant medication that could affect risperidone metabolism (e.g., haloperidol, fluoxetine, paroxetine, carbamazepine, and phenytoin) or prolactin levels (e.g., haloperidol, sertraline, and fluoxetine).

### Study Protocol

The retrospective study included 124 ASD patients treated with a risperidone-based regimen for at least 3 months. Serum prolactin levels, plasma levels of risperidone, 9-OH-risperidone, and the active moiety were measured, and the candidate genes were genotyped. We also included 19 risperidone-naïve patients who underwent a baseline assessment before starting risperidone therapy. They were available for a follow-up assessment 3–20 months after risperidone treatment was started. At the first and follow-up visits, assessments were performed using ABC subscales and serum prolactin and plasma drug levels.

### Behavior Assessments

The ABC subscale assessment consisted of 58 items, divided into the following five categories of behavior: irritability, agitation, and crying (15 items); lethargy and social withdrawal (16 items); stereotypic behavior (7 items); hyperactivity and non-compliance (16 items); and inappropriate speech (4 items). The probands were rated by a primary patient’s caregiver for the different severity of behavior problems from zero (no problems) to three (severe problems), with higher scores indicating problems that were more severe (Narkpongphun and Charnsil, 2018b). The ABC-irritability subscale score is an accepted gold standard for measuring irritability and aggression in medication trials for ASD (Fung et al., 2016). We used the ABC-C Thai version, which was created with a cross-cultural adaptation method, has been validated, and is highly reliable (Narkpongphun and Charnsil, 2018a). Patients with ASD were divided into responders and non-responders according to the reduction rate of the total ABC scores. Patients classified as responders had a reduction rate of total ABC scores higher or equal to 30%, whereas patients with ABC score reduction rates of less than 30% were classified as non-responders.

### Serum Prolactin Measurement

A fasting morning blood sample was analyzed with a chemiluminescent immunoassay system (IMMULITE1000, Siemens Healthcare Diagnostics Products Ltd., Erlangen, Germany) at the Yuwaprasart Waithayopathum Child and Adolescent Psychiatric Hospital, Thailand.

### Plasma Drug Assay

Steady-state plasma concentrations of risperidone and 9-OH-risperidone were quantified, between 8:00 and 10:00 AM, approximately 12 h after the bedtime dose, using a validated high-performance liquid chromatography method (Puangpetch et al., 2016; Vanwong et al., 2016). The active moiety levels were calculated by summing the levels of risperidone and 9-OH-risperidone. Plasma drug levels of risperidone, 9-OH-risperidone, and active moiety were corrected by the daily dose to give the dose-corrected risperidone (RIS C/D), dose-corrected 9-OH-risperidone (9-OH-RIS C/D), and dose-corrected active moiety (active moiety C/D).

### Pharmacogenetic Testing

Genomic DNA was obtained from EDTA blood using the MagNa Pure automated extraction system according to the manufacturer’s instructions (Roche Diagnostics, Basel, Switzerland). The DNA samples were subsequently genotyped for the following sequence variations: *DRD2*/*ANKK1* *Taq*1A A2>A1 (rs1800497), *DRD2* -141C indel (rs1799732), and -241A>G (rs1799978); *HTR2A* -1438G>A (rs6311); *HTR2C* -759C>T (rs3813929); and *PRL* 13096T>A (rs2244502), and *PRLR* 163444A>C (rs37364). SNPs were selected based on functional significance (Kirchheiner et al., 2007; Correia et al., 2010; Porcelli et al., 2012; Outhred et al., 2016; Firouzabadi et al., 2017; Nuntamool et al., 2017) and minor allele frequencies of >0.05 across the Asian population. All SNPs were genotyped using the commercially available TaqMan Drug Metabolism Genotyping assay (Life Technologies, Carlsbad, CA, United States). Genotyping was carried out as recommended by the manufacturer using a real-time PCR system (ViiA7, Applied Biosystems, Life Technologies).

To determine the number of variable tandem repeats (VNTRs) of *DAT*, 60 ng genomic DNA was amplified in 25 μl PCR reactions, containing 2X Green PCR master mix (12.5 μl, BiotechRabbit, Hennigsdorf, Germany), and 1 μl of each 10 μM primer (forward, 5′-TCC​TTG​CGG​TGT​AGG​GAA​CG-3′; reverse 5′-CCA​GGC​AGA​GTG​TGG​TCT​G-3′). Denaturation at 95°C for 2 min was followed by 35 cycles of 95°C for 30 s, 65°C for 40 s, and 72°C for 1 min, and a final extension step at 72°C for 10 min. The fragment sizes were 263 bp (5 repeats), 423 bp (9 repeats), 463 bp (10 repeats), and 503 bp (11 repeats), respectively. For *5-HTTLPR*, 8 µl PCR reactions contained 4 µl KAPA 2G^TM^ Fast ReadyMix (KAPA Biosystems, Woburn, MA, United States), 0.6 μl of each 5 μM primer (forward, 5′-CAC​AAA​CAT​GCT​CAT​TTA​AGA​AGT​G-3′; reverse, 5′-AAA​GGA​AAT​AGC​AGT​GAC​AAG​TTT​G-3′), and 20 ng genomic DNA. PCR was initiated by a 2 min incubation at 95°C, which was followed by 40 cycles of 95°C for 15 s, 62°C for 40 s, and 72°C for 30 s, and a final extension at 72°C for 1 min. Amplicons representing the short (733 bp) and long (777 bp) alleles were separated by 2% agarose gel electrophoreses.

### Genetic Risk Score

We created an algorithm to describe haplotypes with genetic risk scores. For protein expression affecting the prolactin level, the alleles *DRD2 Taq*1A, -141C indel, and -241A>G had values of 1 assigned for a high-expression allele (A2, Cin, G) and 0.5 for a low-expression allele (A1, Cdel, A) (Arinami et al., 1997; Jonsson et al., 1999; Nyman et al., 2009). The score of each haplotype was the sum of the values assigned to each allele. A high risk score indicated a high prolactin level.

### Statistical Analysis

Statistical analyses were carried out using SPSS v24 for Windows (IBM, Armonk, NY, United States). Statistical significance is reported as *p* < 0.05 for a two-tailed distribution. Descriptive analyses were performed for the sociodemographic variables. Data are expressed as mean (standard deviation; SD) or median (interquartile range; IQR) in normal or non-normal distribution data, respectively. Parametric analysis of variance (comparisons between more than two groups) and Student’s *t*-test (comparisons between two groups) were used to assess at each time point the association between prolactin or drug levels and the genotypes. The nonparametric Kruskal-Wallis (comparisons among more than two groups) and Mann-Whitney (comparisons between two groups) tests were used to assess the association between prolactin or drug levels and the genotypes at each time point. The nonparametric Spearman rank correlation test was used to measure the correlation between serum prolactin level and plasma drug level. Because nine variants were tested, the *p*-value significance threshold was adjusted for multiple comparisons. Bonferroni’s correction was applied to adjust for multiple comparisons. According to Bonferroni’s procedure, the corrected *p*-values were calculated by multiplying the *p*-values by 9 for the numbers of variants. The significance threshold of the corrected *p*-values was set as 0.05 (Yi et al., 2014). The Hardy-Weinberg equilibrium, allele, and genotype frequencies of all candidate SNPs were analyzed using Haploview v4.2 (Broad Institute, Cambridge, MA, United States). PHASE v2.1.1 was used to reconstruct haplotype pairs on the same chromosome (Stephens and Scheet, 2005). Fisher’s exact test was used to compare the difference in patient characteristics between responders and non-responders in children and adolescents with ASD. Differences in serum prolactin levels between the *DRD2* diplotypes were assessed by analysis of covariance (ANCOVA), controlling for plasma drug level as a covariate. Receiver operating characteristic (ROC) curves were analyzed and plotted. Performance parameters such as sensitivity, specificity, positive predictive value (PPV), negative predictive value (NPV), and accuracy of the association between serum cut-off value of serum prolactin levels and status of risperidone response were analyzed using MedCalc (https://www.medcalc.org/calc/diagnostic_test.php).

## Results

### Clinical Characteristics

Our sample cohort consisted of 124 children and adolescents with a mean age of 8.81 years (*SD* = 4.04). All patients were diagnosed with ASD and treated with risperidone. Seventy-four patients (59.68%) received risperidone monotherapy. The remaining patients received concomitant medications that did not affect cytochrome P450 2D6 metabolite and prolactin levels. Demographic data are summarized in Table 1.

**TABLE 1:** Patient demographics (*n* = 124).

Clinical information	Median (IQR)
Age (years)	8.00 (5.00–12.00)
Males, *n* (%)	105 (84.68)
Daily risperidone dosage (mg/day)	0.75 (0.50–1.00)
Risperidone treatment duration (months)	37.94 (11.01–94.05)
Risperidone monotherapy, *n* (%)	74 (59.68)
Prolactin level (ng/ml)	15.70 (8.85–22.85)
RIS level (ng/ml)	0.51 (0.14–1.41)
9-OH-RIS level (ng/ml)	5.28 (2.94–9.29)
Active moiety level (ng/ml)	6.11 (3.44–11.63)
Ratio of risperidone/9-OH-RIS	0.09 (0.03–0.21)
RIS C/D (ng/ml/mg)	0.71 (0.22–2.03)
9-OH-RIS C/D (ng/ml/mg)	7.72 (4.94–12.04)
Active moiety C/D (ng/ml/mg)	9.06 (5.82–13.14)

RIS, risperidone; 9-OH-RIS, 9-hydroxy-risperidone; Active moiety, the sum of RIS plus 9-OH-RIS; C/D, dose-corrected concentration; IQR, interquartile range [quartile 1 (Q1) and quartile 3 (Q3)].

### Association Between Genetic Variations, Serum Prolactin Levels, and Response to Risperidone

Genotype frequencies of the tested polymorphisms are shown in Supplementary Table S1. Considering codominant, dominant, and recessive genetic models, there were no differences in serum prolactin levels for *DRD2*/*ANKK1 Taq*1A A2>A1, *DRD2* -141C indel, *DRD2* -241A>G, *HTR2A*-1438G>A, *HTR2C* -759C>T, *PRL* g.13096T>A, *PRLR* g.163444A>C, and the number of variable tandem repeats of *DAT* and *5-HTTLPR*.

Haplotypes were constructed using three SNPs of *DRD2* on chromosome 11 (*DRD2*/*ANKK1 Taq*1A A2/A1, *DRD2* -141C indel, and -241A>G) with PHASE v2.1.1. Six haplotypes with minor allelic frequencies of >1% were identified. The four main haplotypes were A2-Cin-A (H1), A1-Cin-A (H2), A2-Cin-G (H3), and A2-Cdel-A (H4), accounting for 91.4% of the observations (Table 2). Fifteen diplotypes accounted for 99.2% of the observations (Table 3). We compared the association of each pair of diplotype groups. Participants with the H1/H3 diplotype showed significantly higher prolactin levels than those of other diplotypes (23.00 ng/ml, *p* < 0.05).

**TABLE 2:** *DRD2* haplotype frequencies predicted by computational phasing using PHASE v2.1.1.

Type	Haplotype	Observation (*n* = 124)	Frequency (%)
H1	A2-Cin-A	86	34.68
H2	A1-Cin-A	75	30.24
H3	A2-Cin-G	35	14.11
H4	A2-Cdel-A	31	12.50
H5	A1-Cdel-A	11	4.44
H6	A1-Cin-G	10	4.03

Haplotype presented as DRD2/ANKK1 Taq1A, DRD2 -141C indel, and DRD2 -241A>G.

**TABLE 3:** Associations between *DRD2* gene diplotypes and serum prolactin levels.

Types	Diplotypes	Observation (*n* = 124)	Percent (%)	Genetic risk score	Prolactin levels (ng/ml)
H2/H6	A1/A1-Cin/Cin-A/G	4	3.23	4.5	29.40 (15.65–67.70)
H3/H3	A2/A2-Cin/Cin-G/G	3	2.42	6	28.20 (16.00–29.75)
H2/H5	A1/A1-Cin/Cdel-A/A	3	2.42	3.5	25.90 (21.80–31.15)
H1/H3	A2/A2-Cin/Cin-A/G	12	9.68	5.5	23.00 (17.50–35.25)^a,b,c,d^
H3/H5 or H4/H6	A2/A1-Cin/Cdel-A/G	4	3.23	4.5	16.90 (13.15–25.30)
H1/H5 or H2/H4	A2/A1-Cin/Cdel-A/A	17	13.71	4	16.80 (12.15–21.80)^a^
H1/H4	A2/A2-Cin/Cdel-A/A	14	11.29	4.5	16.25 (10.10–24.00)
H3/H4	A2/A2-Cin/Cdel-A/G	2	1.61	5	14.40 (8.00–20.80)
H1/H6 or H2/H3	A2/A1-Cin/Cin-A/G	17	13.71	5	13.30 (7.50–21.40)^b^
H1/H2	A2/A1-Cin/Cin-A/A	23	18.55	4.5	13.00 (7.80–19.40)^c^
H1/H1	A2/A2-Cin/Cin-A/A	14	11.29	5	13.00 (9.10–24.00)
H4/H5	A2/A1-Cdel/Cdel-A/A	1	0.81	3.5	12.40
H2/H2	A1/A1-Cin/Cin-A/A	10	8.06	4	10.60 (7.40–21.80)^d^

Diplotype presented as DRD2/ANKK1 Taq1A, DRD2 -141C indel, and DRD2 -241A>G.

a Significant at *p* = 0.042 when compared between H1/H3 and H1/H5 or H2/H4.

b Significant at *p* = 0.028 when compared between H1/H3 and H1/H6 or H2/H3.

c Significant at *p* = 0.014 when compared between H1/H3 and H1/H2.

d Significant at *p* = 0.038 when compared between H1/H3 and H2/H2.

We created an algorithm to describe a haplotype with a genetic risk score based on *DRD2* expression of protein affecting the prolactin level. Forty-five (36.29%) patients had a common risk score of 4.5 and had a median prolactin level of 16.60 ng/ml. We compared the common risk score and found that prolactin levels in patients with a common genetic risk score of 4.5 (*n* = 45, 36.29%) were significantly lower than those with a genetic risk score of 5.5 (*n* = 12, 9.68%) (16.60 vs. 23.00 ng/ml, *p* = 0.033). Thus, higher *DRD2* expression was related to higher prolactin levels (Table 4). However, there were high prolactin levels at the lowest and highest risk score (21.80 ng/ml at a risk score of 3.5 and 28.20 ng/ml at a risk score of 6, respectively), but there were no significant differences (*p* > 0.05).

**TABLE 4:** Associations between genetic risk scores for *DRD2* gene haplotypes and serum prolactin levels.

Genetic risk score	Diplotypes	Types	N (%) (*n* = 124)	Prolactin levels (ng/ml)	*p*-value
3.5	H2/H5, H4/H5	A1/A1-Cin/Cdel-A/A, A2/A1-Cdel/Cdel-A/A	4 (3.23)	21.80 (15.05–31.15)	0.231
4	H1/H5 or H2/H4, H2/H2	A2/A1-Cin/Cdel-A/A, A1/A1-Cin/Cin-A/A	27 (21.77)	12.70 (8.00–21.60)	0.504
4.5	H1/H2, H1/H4, H2/H6, H3/H5 or H4/H6	A2/A1-Cin/Cin-A/A, A2/A2-Cin/Cdel-A/A, A1/A1-Cin/Cin-A/G, A2/A1-Cin/Cdel-A/G	45 (36.29)	16.60 (9.00–22.70)	Reference
5	H1/H1, H1/H6 or H2/H3, H3/H4	A2/A2-Cin/Cin-A/A, A2/A1-Cin/Cin-A/G, A2/A2-Cin/Cdel-A/G	33 (26.61)	13.00 (8.00–21.40)	0.498
5.5	H1/H3	A2/A2-Cin/Cin-A/G	12 (9.68)	23.00 (17.50–35.25)	0.033^a^
6	H3/H3	A2/A2-Cin/Cin-G/G	3 (2.42)	28.20 (16.00–29.75)	0.413

Diplotype presented as DRD2/ANKK1 Taq1A, DRD2 -141C indel, and DRD2 -241A>G respectively as follow: high expression allele (A2, Cin, G) = 1 and low expression allele (A1, Cdel, A) = 0.5. A high-risk score assumed a high prolactin level.

a Significant at *p* < 0.05.

There was no association between any of the interrogated sequence variations in the candidate pharmacodynamic genes and the risperidone response (Supplementary Table S2).

### Relationships Between Serum Prolactin Levels and Plasma Levels of RIS C/D, 9-OH-RIS C/D, and Active Moiety C/D

Plasma levels of risperidone, 9-OH-risperidone, and the active moiety were corrected with the daily dose. Serum prolactin levels were significantly correlated among RIS C/D (*r*_s_ = 0.227, *p* = 0.012), 9-OH-RIS C/D (*r*_s_ = 0.305, *p* = 0.001), and active moiety C/D (*r*_s_ = 0.343, *p* < 0.001).

### Relationships Between Serum Prolactin, Plasma RIS C/D, 9-OH-RIS C/D, Active Moiety C/D and Sequence Variations in Candidate Pharmacodynamic Genes

**FIGURE 1:**
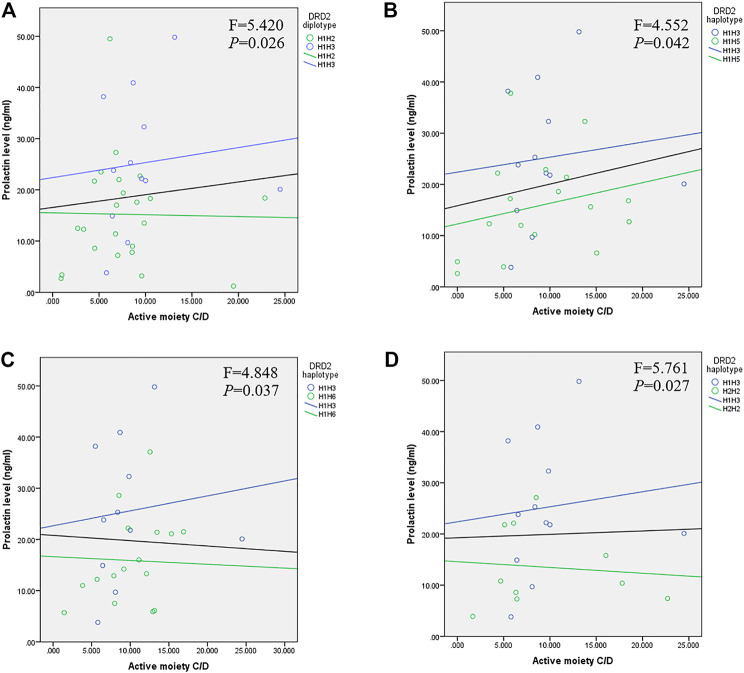
Relationship between prolactin and active moiety C/D plasma levels in children and adolescents with *DRD2* diplotypes of **(A)** H1/H3 and H1/H2 (*p* = 0.026), **(B)** H1/H3 and H1/H5 (*p* = 0.042), **(C)** H1/H3 and H1/H6 (*p* = 0.037), and **(D)** H1/H3 and H2/H2 (*p* = 0.027). The black line represents the linear relationship between prolactin and drug levels in patients carrying an H1/H3 or other diplotype.

We conducted ANCOVA to compare the effect of *DRD2* diplotypes on prolactin levels, using RIS C/D, 9-OH-RIS C/D, and active moiety C/D as covariates to adjust for possible confounding factors. Serum levels of prolactin were significantly higher in the H1/H3 diplotype group compared with the H1/H2 (Figure 1A; F = 5.420, *p* = 0.026), H1/H5 (Figure 1B; F = 4.552, *p* = 0.042), H1/H6 (Figure 1C; F = 4.848, *p* = 0.037), and H2/H2 (Figure 1D; F = 5.761, *p* = 0.027) groups after controlling for plasma drug levels of active moiety C/D.

### Effects of Prolactin in Response to Risperidone Therapy

A total of 124 participants were enrolled in this study; however, only 19 patients were available for naïve and follow-up after being treated for a minimum of 3 months. The mean age at baseline of the 19 patients was 5.21 years (SD 2.82), and most were male (*n* = 16, 84.21%). Table 5 summarizes the findings for the 19 patients by responder status according to the percentage decrease in the total ABC score. Of these, 53% (*n* = 10) responded to risperidone treatment, whereas 47% (*n* = 9) were non-responders. The mean age of responders was 5.50 ± 2.84 years and that of non-responders was 4.89 ± 2.93 years. Median prolactin levels after treatment in non-responders were higher compared with those in responders (20.10 vs. 10.25 ng/ml, *p* = 0.013). ABC subscale scores after 3 months were significantly lower than those before treatment (paired *t*-test; *p* < 0.05). Plasma levels of RIS C/D, 9-OH-RIS C/D, and active moiety C/D were not significantly different between responders and non-responders. Moreover, there was no significant difference in prolactin levels before and after treatment in responders (7.65 vs. 10.25 ng/ml, *p* = 0.878). In contrast, the prolactin level in non-responders after risperidone treatment was about twice that in responders (9.40 vs. 20.10 ng/ml, *p* = 0.028).

**TABLE 5:** ABC score and serum prolactin levels at baseline and after 3 months of treatment between responders and non-responders (*n* = 19).

Variables	Responders *(n =* 10*)*	Non-responders *(n =* 9*)*	*p-*value
Male, *n* (%)	10 (100)	6 (66.67)	0.087
Age (years), median (IQR)	4.50 (4.00–6.00)	5.00 (4.00–7.00)	0.905
Baseline			
Body weight (kg), median (IQR)	17.58 (15.70–25.00)	20.00 (15.30–27.00)	0.968
Risperidone dose (mg/day), median (IQR)	0.20 (0.20–0.50)	0.25 (0.15–0.50)	0.842
Weight-adjusted dose (mg/kg day), median (IQR)	0.01 (0.01–0.01)	0.02 (0.01–0.02)	0.315
ABC total score, mean ± SD	85.70 ± 29.73	91.22 ± 26.29	0.675
ABC-irritability, mean ± SD	20.40 ± 8.17	21.89 ± 11.42	0.746
ABC-social withdrawal, mean ± SD	16.80 ± 9.31	22.78 ± 6.80	0.132
ABC-stereotype, mean ± SD	9.50 ± 6.08	9.56 ± 5.64	0.984
ABC-hyperactivity, mean ± SD	33.10 ± 8.90	32.44 ± 5.88	0.854
ABC-inappropriate speech, mean ± SD	5.90 ± 4.12	4.56 ± 3.32	0.448
Prolactin level (ng/ml), median (IQR)	7.65 (6.00–17.70)	9.40 (7.10–16.60)	1.000
After 3 months			
Body weight (kg), median (IQR)	20.18 (17.00–30.00)	23.60 (16.70–32.00)	0.905
Risperidone dose (mg/day), median (IQR)	0.50 (0.20–0.60)	0.30 (0.20–0.50)	0.315
Weight-adjusted dose (mg/kg day), median (IQR)	0.02 (0.01–0.03)	0.01 (0.01–0.02)	0.356
Risperidone duration (months), median (IQR)	8.64 (3.00–13.77)	4.37 (3.70–7.03)	0.780
ABC total score, mean ± SD	41.50 ± 18.00	84.67 ± 14.63	<0.001
ABC-irritability, mean ± SD	7.90 ± 5.26	20.78 ± 11.55	0.011
ABC-social withdrawal, mean ± SD	8.20 ± 3.94	19.89 ± 6.90	<0.001
ABC-stereotype, mean ± SD	3.40 ± 2.68	7.78 ± 5.14	0.041
ABC-hyperactivity, mean ± SD	18.60 ± 8.21	31.44 ± 4.98	0.001
ABC-inappropriate speech, mean ± SD	3.40 ± 2.91	4.89 ± 3.55	0.330
Prolactin level, median (IQR), ng/ml	10.25 (6.50–16.00)	20.10 (15.80–27.40)	0.013
Risperidone, median (IQR), ng/ml	0.19 (0.02–0.90)	0.33 (0.12–0.58)	0.720
9-OH-RIS, median (IQR), ng/ml	3.04 (1.67–5.26)	4.57 (3.26–7.27)	0.400
Active moiety, median (IQR), ng/ml	3.86 (1.67–5.56)	5.41 (3.28–8.02)	0.447
Risperidone C/D, median (IQR), ng/ml	23.56 (3.17–40.30)	7.19 (3.55–36.12)	0.624
9-OH-RIS C/D, median (IQR), ng/ml	9.68 (7.12–18.64)	8.77 (6.52–15.09)	0.935
Active moiety C/D, median (IQR), ng/ml	13.58 (9.06–19.42)	9.17 (6.77–16.90)	0.638

RIS, risperidone; 9-OH-RIS, 9-hydroxy-risperidone; Active moiety, the sum of RIS plus 9-OH-RIS; C/D, dose-corrected concentration; IQR, interquartile range [quartile 1 (Q1) and quartile 3 (Q3)]; SD, standard deviation.

### ROC Curve of Serum Prolactin for Responders

**FIGURE 2:**
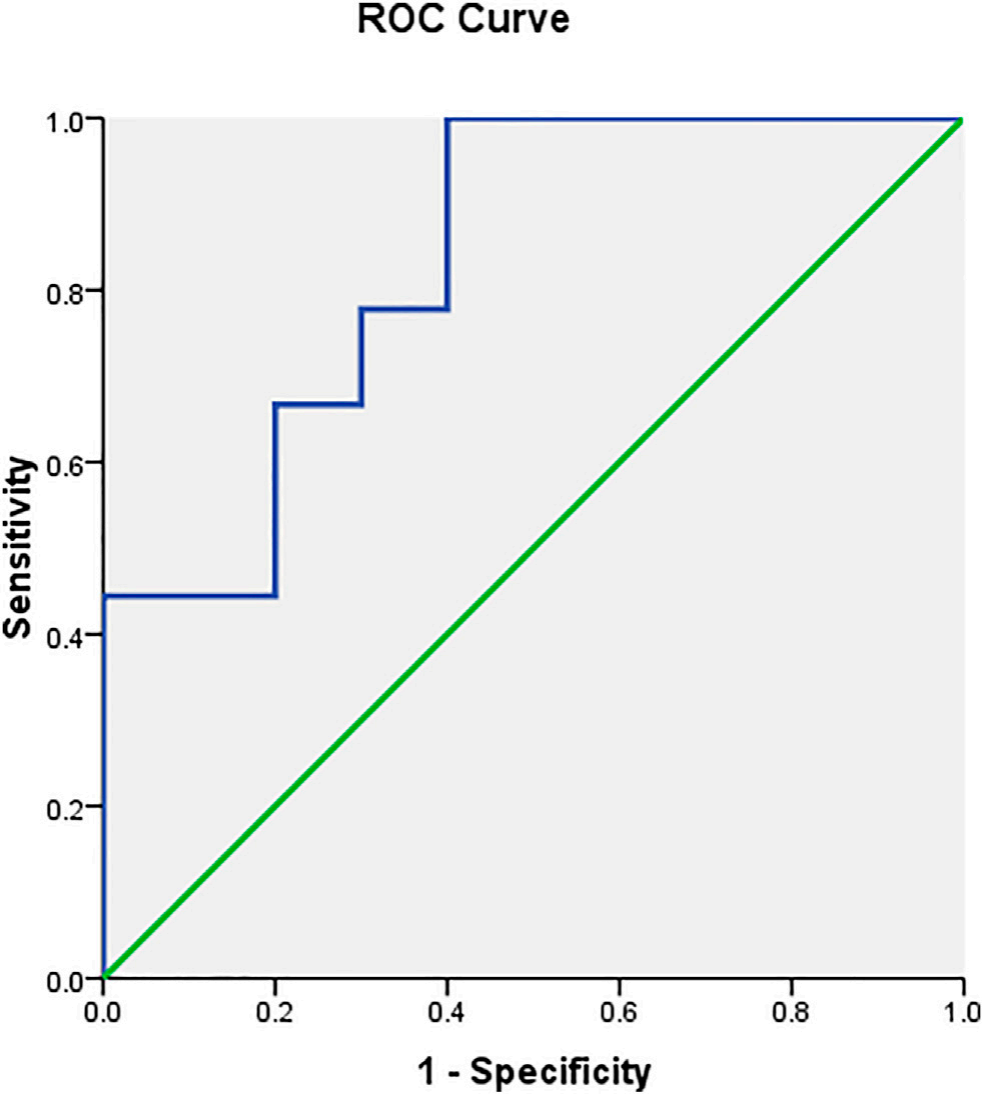
ROC curve analysis showing that serum prolactin level has a high accuracy (68–78%) for identifying responders and non-responders.

The ROC curve showed that serum prolactin levels predicted the risperidone response (Figure 2 and Table 6). The area under the ROC curve for prolactin was 0.833 (*p* = 0.014), which was above the acceptable accuracy level of 0.8. The prolactin cut-off value with sensitivity and specificity of 0.5 or greater was 10.25–18.85 ng/ml, with an optimal prolactin cut-off value of 10.9 ng/ml (sensitivity, 100%; specificity, 60%; PPV, 69.23%; NPV, 100.00%; highest accuracy, 78.95%). The second accuracy for serum prolactin levels of 10.25, 12.20, 15.0, and 16.8 ng/ml was 73.68%.

**TABLE 6:** Sensitivity, specificity, and accuracy of prolactin levels predicting risperidone response.

Prolactin levels (ng/ml)	Sensitivity (%)	Specificity (%)	PPV (%)	NPV (%)	Accuracy (%)
1.70	100.00	0.00	47.37	-*	47.37
3.30	100.00	10.00	50.00	100.00	52.63
5.20	100.00	20.00	52.94	100.00	57.89
6.55	100.00	30.00	56.25	100.00	63.16
8.35	100.00	40.00	60.00	100.00	68.42
**10.25**	100.00	50.00	64.29	100.00	73.68
***10.90***	100.00	60.00	69.23	100.00	78.95
**12.20**	88.89	60.00	66.67	85.71	73.68
**13.60**	77.78	60.00	63.64	75.00	68.42
**15.00**	77.78	70.00	70.00	77.78	73.68
**15.90**	66.67	70.00	66.67	70.00	68.42
**16.80**	66.67	80.00	75.00	72.73	73.68
**18.85**	55.56	80.00	71.43	66.67	68.42
20.80	44.44	80.00	66.67	61.54	63.16
21.85	44.44	90.00	80.00	64.29	68.42
23.00	44.44	100.00	100.00	66.67	73.68
25.60	33.33	100.00	100.00	62.50	68.42
28.80	22.22	100.00	100.00	58.82	63.16
30.45	11.11	100.00	100.00	55.56	57.89
31.70	0.00	100.00	-*	52.63	52.63

PPV, positive predictive value; NPV, negative predictive value.

Bold values represented cut-off value associated with higher and equal 50% in both sensitivity and specificity.

The italicized value represented the cut-off value associated with higher and equal 50% in both sensitivity and specificity with the highest degree of accuracy.

*PPV or NPV cannot be estimated.

## Discussion

Several studies have reported that genetic variation (Young et al., 2004; Calarge et al., 2009; Yasui-Furukori et al., 2011) or plasma drug levels of risperidone and its metabolite (Knegtering et al., 2005; Melkersson, 2006; Ngamsamut et al., 2016) are related to hyperprolactinemia. However, there are no studies on the relationship between prolactin elevation and genetic variation, or whether controlling plasma drug levels of RIS C/D, 9-OH-RIS C/D, and active moiety C/D can prevent prolactin elevation. The findings of the present study suggest that the patients with the H1/H3 haplotype of *DRD2* were at higher risk than patients with other diplotypes of presenting with significantly higher prolactin levels with increasing plasma RIS C/D, 9-OH-RIS C/D, and active moiety C/D. Furthermore, low prolactin levels were associated with response to risperidone treatment in Thai ASD children and adolescents.

Hyperprolactinemia is commonly observed in patients treated with antipsychotics. In our study, 44.35% (55 of 124) of patients had high prolactin levels, which is a concern for adverse drug reactions in children and adolescents treated with risperidone. The reported prevalence of hyperprolactinemia in patients on risperidone ranges between 44 and 61% (Hongkaew et al., 2015; An et al., 2016; Bonete Llacer et al., 2019), and abnormal prolactin levels occur in about 27% of patients taking risperidone (Lally et al., 2017). Hypothalamic dopamine inhibits prolactin secretion by acting on dopamine D2 receptors, which inhibits lactotroph cells (Ben-Jonathan and Hnasko, 2001). Hypothalamic dopamine inhibits pituitary prolactin secretion and proliferation of prolactin-producing lactotroph cells by activating lactotroph dopamine D2 receptors (Ben-Jonathan and Hnasko, 2001; Fitzgerald and Dinan, 2008). There are several hypotheses for why prolactin elevation occurs during treatment with certain antipsychotics. Risperidone may cause high prolactin levels because of its slow dissociation from dopamine D2 receptors, which increases central dopamine D2 receptor occupancy (Peuskens et al., 2014), resulting in a prolonged blockade and higher rates of prolactin release. Risperidone is also associated with weaker blood-brain barrier penetration (Peuskens et al., 2014) compared with olanzapine and quetiapine (Arakawa et al., 2010), resulting in greater dopamine D2 receptor occupancy in the pituitary area, which also increases prolactin levels.

We also investigated whether *DRD2* haplotypes are more informative than each SNP on its own, and the H1/H3 diplotype had the highest serum prolactin levels. These findings are consistent with increased prolactin levels in patients carrying the *DRD2*/*ANKK1 Taq*1A A2 or C allele (Sukasem et al., 2016). These alleles are associated with high dopamine receptor densities (Jonsson et al., 1999), which may contribute to blocking dopaminergic activity. *DRD2* -241A>G (rs1799978), which is located in the 5′-promoter region of *DRD2*, may contribute to increased expression levels. Data obtained with lymphoblastoid cell lines suggested that -241G carriers have higher *DRD2* expression levels than non-carriers (Nyman et al., 2009), which is consistent with our conclusion that *DRD2* -241A>G is involved in hyperprolactinemia. Our results are also consistent with previous findings. Calarge et al. (2009) found that the -241G allele had the largest effect on serum prolactin levels when combined with the *Taq*1A A1 allele, whereas Arinami et al. (1997) reported an association between the *DRD2* -141C indel and high *DRD2* expression levels *in vitro*. Stronger binding of risperidone to dopamine D2 receptors in the pituitary area is more likely to inhibit dopaminergic activity in prolactin secretion (Peuskens et al., 2014). Therefore, the findings of these studies support our observation that increased *DRD2* expression levels, mediated by the *DRD2* -141C indel, contribute to the increase in prolactin levels. Our finding is also consistent with a study describing antipsychotic-induced sexual dysfunction in male schizophrenia, which was attributed to high prolactin levels in the presence of the *DRD2* -141C indel (Zhang et al., 2011). Therefore, the synergistic effects of the *DRD2*/*ANKK1 Taq*1A A2>A1, *DRD2* -141C indel, and -241A>G SNPs may be better captured by the association of haplotypes, rather than SNPs, with serum prolactin levels. Based on our results, we suggest that clinicians should perform DNA analysis of the three SNPs of the *DRD2* haplotype as pre-emptive genetic testing, and prolactin levels should be monitored before and after 3 months to prevent hyperprolactinemia, especially in patients with the H1/H3 diplotype.

We developed an algorithm to categorize *DRD2* haplotypes simply according to their *DRD2* expression (Arinami et al., 1997; Jonsson et al., 1999; Nyman et al., 2009). High-expression alleles (A2, Cin, G) were assigned a score of 1 and low-expression alleles (A1, Cdel, A) were assigned a score of 0.5. The score of each haplotype was the sum of the values of each allele. A high risk score was expected to indicate a high prolactin level. In this study, a higher *DRD2* expression score was significantly related to a higher prolactin level. The increase in prolactin levels with risperidone can be explained by risperidone inhibiting dopamine binding to dopamine D2 receptors (Rosenbloom, 2010). The higher the number of dopamine receptors is, the greater the inhibition by risperidone, and the higher the increase in prolactin. Thus, high prolactin levels occurred at the highest risk score of 6. However, this study also observed the high prolactin level at the lowest risk score of 3.5. This could be explained by the upregulation of dopamine receptors in patients with low *DRD2* expression and who received long-term risperidone treatment. Lidow and Goldman-Rakic (1997) found that 6 months treatment with antipsychotics upregulated prefrontal and temporal cortical dopamine D2 receptor mRNA expression in primates. However, there was no significant difference in expression due to the small sample size. The genetic risk score system is an easy-to-use tool for translating genotype data into *DRD2* expression prediction in a clinical setting. However, further study is needed to validate this tool.

Because variations in the *HTR2A* gene (encoding the 5-hydroxytryptamine type 2A receptor) and *HTR2C* gene (encoding the 5-hydroxytryptamine type 2C receptor) influence the binding affinities of antipsychotic medication, in previous studies, two genetic polymorphisms related to hyperprolactinemia in schizophrenic patients treated with classical and/or atypical antipsychotic treatment have been analyzed. Several studies found contradictory associations between hyperprolactinemia and *HTR2A* (Correia et al., 2010; Ivanova et al., 2017) and *HTR2C* polymorphisms (Alladi et al., 2017; Koller et al., 2020). Our study was consistent with a study of risperidone in 289 Indian schizophrenia patients (Alladi et al., 2017). They reported no association between *HTR2C* (−759 C>T) genetic variants and prolactin levels during risperidone treatment. Genetic polymorphisms in the promoter region of the *HTR2C* gene (−759 C>T) have been investigated in antipsychotic drug-induced weight gain (Vanwong et al., 2021). Moreover, transporter and prolactin-related genes involving the neurotransmitter mechanism of prolactin secretion were studied. Osmanova et al. (2019) found an association between two variants in the *DAT* (*SLC6A3*) gene and hyperprolactinemia in the subgroup of patients taking risperidone/paliperidone in 446 Caucasian schizophrenia patients. Smith et al. (2004) reported that the short allele of the *5-HTTLPR* indel polymorphism was associated with less of an increase in prolactin and cortisol than the long/long genotype in control participants. Lee et al. (2007) found a nominally significant association between *PRL* and *PRLR* tagSNPs and plasma prolactin levels in 95 advanced breast cancer cases. However, the results were inconsistent because of the different underlying disease groups and treatments. Thus, until our results are validated in a prospective study, they should not be applied to patients with other diseases or undergoing other treatments.

There were no significant differences between any genetic variants selected and responses to risperidone in this study. Thus, although the *DRD2* haplotypes relate to prolactin increase, they are likely to be predictive for poor markers for treatment response. Our results agree with Sakumoto et al. (2007), who found that non-responders without the deletion allele of the *DRD2* -141C indel showed a higher score for psychiatric, extrapyramidal, and total side-effects than those with the deletion allele after 3 weeks of treatment with dopamine antagonists. The small number of 19 patients in our study cohort may explain why there were no significant differences among the associations between genetic variants and treatment response.

Several studies have examined optimizing risperidone dosing in children and adolescents based on their clinical response (Research Units on Pediatric Psychopharmacology Autism, 2005; Haas et al., 2009; Pozzi et al., 2016). We found no significant association between plasma drug levels and risperidone response due to the small sample size, although non-responders had numerically higher drug plasma levels than responders, which is consistent with other published work (Wang et al., 2007; Lostia et al., 2009). However, our results contradict other studies that show a relationship between therapeutic drug levels and clinical efficacy (Baumann et al., 2004; Riedel et al., 2005). This discrepancy may be a result of the different underlying disease in patients with schizophrenia. In addition, there may be differences in the timing of the clinical outcome assessment and the sociodemographic profile of the patient population among studies. We performed our assessment at least 3 months into risperidone treatment rather than at 1–6 weeks because prolactin levels in children receiving long-term risperidone tend to peak within the first 2 months, and then steadily decline to values within or close to normal after 3–5 months (Findling et al., 2003). Although we did not discover an association between plasma drug concentration and efficacy, the association between plasma drug and serum prolactin levels remained significant. Therefore, monitoring risperidone plasma levels may help to prevent adverse drug reactions, such as hyperprolactinemia.

Although other studies have found that prolactin levels are inconsistently associated with efficacy (Wang et al., 2007; Lostia et al., 2009), our study revealed that prolactin levels may induce changes in neurogenesis, potentially affecting aberrant behaviors in ASD, consistent with several other studies (Zhang et al., 2002; Lee and Kim, 2006; Ates et al., 2015). Prolactin is an anterior pituitary peptide hormone, under inhibitory control by dopamine released from the tuberoinfundibular dopaminergic neurons (Grattan, 2015). Because of dopamine regulation of serum prolactin levels through dopamine D2 receptors in the hypothalamic-pituitary pathway, the serum prolactin levels may be a marker of central dopamine function. Risperidone could induce hyperprolactinemia in excessive dopamine receptor blockade (Oda et al., 2015) due to its high dopamine D2 receptor occupancy (68–70%) (Tsuboi et al., 2013), resulting in hyperprolactinemia in early treatment. Long-term treatment with antipsychotics could lead to increased dopamine D2 receptor density according to the dopamine supersensitivity psychosis hypothesis (Oda et al., 2015), and thus could cause adequate blockade, leading to behavioral improvement. This hypothesis may explain why some ASD patients had increased prolactin levels early in risperidone treatment and subsequently presented with improved symptoms.

The association between high prolactin and worse response could be explained by the brain/plasma concentration ratio of risperidone. Arakawa et al. (2010) reported that risperidone showed a slightly higher brain/plasma concentration ratio. The brain/plasma concentration ratio was calculated from dopamine D2 receptor occupancy in the temporal cortex and pituitary, which represented the permeability of antipsychotics into the brain. The brain/plasma concentration ratio of risperidone was 1.61 (Arakawa et al., 2010), indicating that the risperidone concentration in the temporal cortex was 1.5-fold greater than that in the pituitary. The permeability of risperidone into the brain resulted in a higher concentration in the brain than in plasma. Dopamine D2 receptor occupancy of risperidone in the temporal cortex was higher than that in the pituitary, causing an improvement in behaviors and reducing the risk of prolactin elevation. Therefore, our results suggest that low prolactin levels are an indicator of response to risperidone, and thus serum prolactin levels could be used as an indirect biomarker for risperidone response.

The present study is the first to report a cut-off value for prolactin-related poor behavioral responses of children treated with risperidone of less than 1 mg/day. The prediction of risperidone response in ASD had an area under the ROC curve of 0.833. The cut-off point of the prolactin level associated with sensitivity and specificity of 0.5 or higher was 10.25–18.85 ng/ml, and the optimal cut-off value for prolactin of 10.9 ng/ml had the highest accuracy of 78.95%. We applied the prolactin measurement as a biomarker for unusual behaviors in patients who did not respond to risperidone treatment. It might be used as a screening test to exclude false negatives. When NPV was 100%, meaning that when the test was negative (low prolactin value), the patient was responding to treatment, when the PPV was 60%, meaning that when the test was positive (high prolactin value) of 100 patients, only 60 patients were not responding to treatment. This information would be beneficial to health practitioners in clinical practice. However, further studies are needed to determine the optimal daily dose of risperidone for prolactin monitoring.

There are several limitations to this study. The small sample size in the cohort study prevented us from detecting significant genotype-phenotype associations, and thus the results should be viewed with caution. No relationship was observed for risperidone dose, weight-adjusted dose, and treatment duration between responders and non-responders. However, the study used cohorts under clinical conditions and did not control for risperidone dose and treatment duration.

## Conclusion

We created a system for representing the expression of *DRD2* haplotypes using genetic risk scores. Our findings suggest that patients with the H1/H3 diplotype, which had a *DRD2* genetic risk score of 5.5, showed the highest serum prolactin levels, correlated with increasing plasma RIS C/D, 9-OH-risperidone C/D, and active moiety C/D. Lower levels of prolactin were detected in patients who responded to risperidone. These findings further our understanding of hyperprolactinemia, which is a commonly observed adverse effect of risperidone treatment. Preemptive pharmacogenetic testing may allow clinicians to identify patients at risk of developing high prolactin levels at the outset of therapy and use this information to guide therapeutic decision-making.

## Data Availability

The original contributions presented in the study are included in the article/Supplementary Material, further inquiries can be directed to the corresponding author.
