# Supplementary material for: Pharmacogenomics Factors Influencing the Effect of Risperidone on Prolactin Levels in Thai Pediatric Patients With Autism Spectrum Disorder
Source: Front Pharmacol. 2021 Oct 6;12:743494. doi: 10.3389/fphar.2021.743494 (PMC8527557; doi:10.3389/fphar.2021.743494)
Supplement: Supplementary file 1 [file DataSheet1.docx]

**Supplementary Table 1 Association between candidate SNPs in pharmacodynamic genes and serum prolactin levels (n=124)**

| **Gene** | **Genotype** | **n (%)** | **Prolactin level (ng/ml)^a^** | **p-value** | **Corrected p-value^b^** |
| --- | --- | --- | --- | --- | --- |
| *DRD2*  Taq1A A2>A1  rs1800497 | A2/A2 | 45 (36.29) | 16.60 (9.70-25.30) | 0.382 | 3.438 |
|  | A2/A1 | 62 (50.00) | 13.70 (8.60-21.40) |  |  |
|  | A1/A1 | 17 (13.71) | 17.70 (8.60-27.10) |  |  |
| *DRD2*  -141C indel  rs1799732 | Cin/Cin | 83 (66.94) | 14.90 (8.60-23.10) | 0.908 | 8.172 |
|  | Cin/Cdel | 40 (32.26) | 16.70 (10.65-22.55) |  |  |
|  | Cdel/Cdel | 1 (0.81) | 12.40 |  |  |
| *DRD2*  -241A>G  rs1799978 | A/A | 82 (66.13) | 13.65 (8.60-22.00) | 0.149 | 1.341 |
|  | A/G  G/G | 39 (31.45)  3 (2.42) | 20.10 (11.60-26.35)  28.20 (16.00-29.75) |  |  |
| *HTR2A*  -1438G>A  rs6311 | G/G | 10 (8.06) | 17.35 (11.00-25.80) | 0.747 | 6.723 |
|  | G/A | 46 (37.10) | 16.20 (8.60-23.50) |  |  |
|  | A/A | 68 (54.84) | 15.25 (8.95-22.20) |  |  |
| *HTR2C*  -759C>T  rs3813929 | C/C | 89 (71.77) | 16.60 (10.10-23.80) | 0.353 | 3.177 |
|  | C/T | 9 (7.26) | 13.30 (12.40-22.20) |  |  |
|  | T/T | 26 (20.97) | 12.70 (6.60-21.10) |  |  |
| *PRL*  13096T>A  rs2244502 | T/T | 19 (15.32) | 15.70 (8.70-22.20) | 0.678 | 6.102 |
|  | T/A | 45 (36.29) | 17.20 (11.00-27.10) |  |  |
|  | A/A | 60 (48.39) | 13.80 (7.65-26.30) |  |  |
| *PRLR*  163444A>C  rs37364 | A/A | 73 (58.87) | 17.00 (10.10-22.90) | 0.207 | 1.863 |
|  | A/C | 50 (40.32) | 13.45 (8.00-22.20) |  |  |
|  | C/C | 1 (0.81) | 37.70 |  |  |
| *DAT* VNTR | 11R/10R  (High number of repeat) | 3 (2.46) | 18.30 (13.05-21.15) | 0.470 | 4.23 |
|  | 10R/10R | 87 (71.31) | 14.20 (8.60-21.90) |  |  |
|  | 9R/10R  9R/9R  5R/10R  (Low number of repeat) | 32 (26.23) | 16.80 (10.55-25.55) |  |  |
| *5-HTTLPR* | 16R/16R  (long/long) | 12 (9.84) | 13.00 (11.25-30.00) | 0.655 | 5.895 |
|  | 14R/16R (short/long) | 47 (38.52) | 14.20 (5.45-23.10) |  |  |
|  | 14R/14R (short/short) | 57 (46.72) | 16.80 (10.40-22.20) |  |  |
|  | 16R/20R  14R/20R  14R/22R (extra-long carriers) | 6 (4.92) | 15.50 (13.50-23.80) |  |  |

^a^Values expressed as median (interquartile range)

^b^P-value after Bonferroni’s correction for multiple comparisons.

*DRD2*, dopamine D2 receptor gene; *HTR2A*, 5-hydroxytryptamine 2A receptor gene; *HTR2C*, 5-hydroxytryptamine 2C receptor gene; *PRL*, prolactin gene; *PRLR*, prolactin receptor gene; DAT VNTR, dopamine transporter variable number of tandem repeat polymorphism; *5-HTTLPR*, serotonin-transporter-linked polymorphic region.

**Supplementary Table 2 Relationship between interrogated variants and risperidone response**

| Variants | N | Responders (n=10)  n (%) | Non-responders (n=9)  n (%) | p-value | 95% CI |
| --- | --- | --- | --- | --- | --- |
| *DRD2*  Taq1A A2>A1  rs1800497 |  |  |  |  |  |
| A2/A2 | 6 | 1 (16.67) | 5 (83.33) | 0.057 | 0.008-1.029 |
| A2/A1+A1/A1 | 13 | 9 (69.23) | 4 (30.77) |  |  |
| *DRD2*  -141C indel  rs1799732 |  |  |  |  |  |
| C/C | 15 | 7 (46.67) | 8 (53.33) | 0.582 | 0.024-3.483 |
| C/- + -/- | 4 | 3 (75.00) | 1 (25.00) |  |  |
| *DRD2*  -241A>G  rs1799978 |  |  |  |  |  |
| A/A | 12 | 6 (50.00) | 6 (50.00) | 1.000 | 0.115-4.898 |
| A/G+G/G | 7 | 4 (57.14) | 3 (42.86) |  |  |
| *HTR2A*  -1438G>A  rs6311 |  |  |  |  |  |
| G/G | 9 | 4 (44.44) | 5 (55.56) | 0.656 | 0.086-3.307 |
| G/A+A/A | 10 | 6 (60.00) | 4 (40.00) |  |  |
| *HTR2C*  -759C>T  rs3813929 |  |  |  |  |  |
| C/C | 14 | 7 (50.00) | 7 (50.00) | 1.000 | 0.084-5.301 |
| C/T+T/T | 5 | 3 (60.00) | 2 (40.00) |  |  |
| *PRL*  13096T>A  rs2244502 |  |  |  |  |  |
| T/T | 11 | 7 (63.64) | 4 (36.36) | 0.370 | 0.442-19.234 |
| T/A+A/A | 8 | 3 (37.50) | 5 (62.50) |  |  |
| *PRLR*  163444A>C  rs37364 |  |  |  |  |  |
| A/A | 8 | 4 (50.00) | 4 (50.00) | 1.000 | 0.134-5.167 |
| A/C+C/C | 11 | 6 (54.55) | 5 (45.45) |  |  |
| *DAT* VNTR |  |  |  |  |  |
| 10R/10R | 13 | 9 (69.23) | 4 (30.77) | 0.057 | (0.972-130.221) |
| Low number of repeat (9R/10R, 9R/9R, 5R/10R) | 6 | 1 (16.67) | 5 (83.33) |  |  |
| *5-HTTLPR* |  |  |  |  |  |
| 14R/14R | 10 | 5 (50.00) | 5 (50.00) | 0.809 | 0.131-4.874 |
| 14R/16R+16R/16R | 9 | 5 (55.56) | 4 (44.44) |  |  |

*DRD2*, dopamine D2 receptor gene; *HTR2A*, 5-hydroxytryptamine 2A receptor gene; *HTR2C*, 5-hydroxytryptamine 2C receptor gene; *PRL*, prolactin gene; *PRLR*, prolactin receptor gene; *DAT* VNTR, dopamine transporter variable number of tandem repeat polymorphism; *5-HTTLPR*, serotonin-transporter-linked polymorphic region; CI, confidential interval
